# TreeGrafter: phylogenetic tree-based annotation of proteins with Gene Ontology terms and other annotations

**DOI:** 10.1093/bioinformatics/bty625

**Published:** 2018-07-19

**Authors:** Haiming Tang, Robert D Finn, Paul D Thomas

**Affiliations:** 1 Division of Bioinformatics, Department of Preventive Medicine, University of Southern California, Los Angeles, CA, USA; 2 European Molecular Biology Laboratory, European Bioinformatics Institute (EMBL-EBI), Wellcome Trust Genome Campus, Hinxton, Cambridge, UK

## Abstract

**Summary:**

TreeGrafter is a new software tool for annotating protein sequences using pre-annotated phylogenetic trees. Currently, the tool provides annotations to Gene Ontology (GO) terms, and PANTHER family and subfamily. The approach is generalizable to any annotations that have been made to internal nodes of a reference phylogenetic tree. TreeGrafter takes each input query protein sequence, finds the best matching homologous family in a library of pre-calculated, pre-annotated gene trees, and then grafts it to the best location in the tree. It then annotates the sequence by propagating annotations from ancestral nodes in the reference tree. We show that TreeGrafter outperforms subfamily HMM scoring for correctly assigning subfamily membership, and that it produces highly specific annotations of GO terms based on annotated reference phylogenetic trees. This method will be further integrated into InterProScan, enabling an even broader user community.

**Availability and implementation:**

TreeGrafter is freely available on the web at https://github.com/pantherdb/TreeGrafter, including as a Docker image.

**Supplementary information:**

Supplementary data are available at *Bioinformatics* online.

## 1 Introduction

The growing rate of new protein sequence discovery continues to increase the demand for automated computational methods for functionally annotating these sequences. The Gene Ontology (GO) is by far the most highly used, computationally accessible representation of gene and protein function (Ashburner *et al.*, 2000; The Gene Ontology Consortium, 2017). Several methods have been developed to infer GO annotations for experimentally uncharacterized protein sequences. Blast2GO finds homologs of input sequences using BLAST, extracts existing GO annotations for obtained hits, and finally assigns GO terms for query sequences using an annotation rule (Conesa *et al.*, 2005). InterPro2GO (Burge *et al.*, 2012) associates GO terms with InterPro entries, and propagates GO terms to sequences based on matching InterPro entries (Mitchell *et al.*, 2015). PANTHER (Mi *et al.*, 2017) classifies sequences using two types of hidden Markov model (HMM): family HMMs (that recognize members of a large family tree) and subfamily HMMs (that recognize members of a sub-family within the family tree) and similarly annotates the query sequence with the GO annotations of the matching HMMs.

Over the past few years, biocurators in the GO Consortium have annotated over 5000 gene trees with GO terms using the Phylogenetic Annotation and INference Tool (PAINT) (Gaudet *et al.*, 2011). These annotations are based on experimental GO annotations, and consider each GO term on a case-by-case basis, decreasing false positive and false negative function prediction rates (Gaudet *et al.*, 2011). PAINT has been used to annotate protein sequences from the ∼100 genomes in these reference trees, but until now there has been no way to apply these annotations to the millions of sequences uncovered by other sequencing projects, both whole genome and metagenome.

**Fig. 1.**
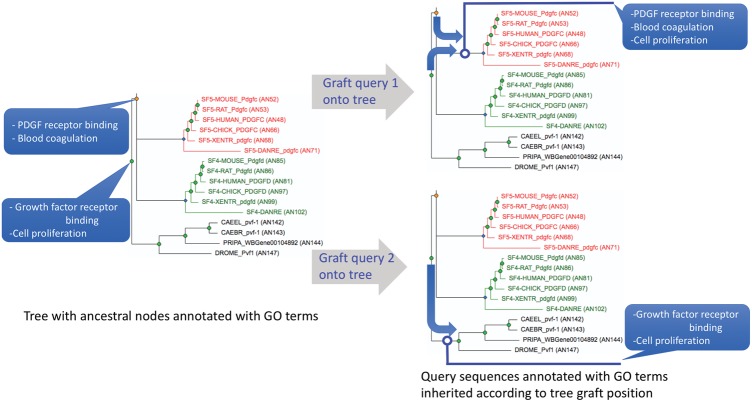
TreeGrafter annotates each sequence based on where it is grafted onto an annotated reference tree. Given the same tree with pre-annotated ancestral gene nodes (left panel), each query sequence is grafted onto the tree. For the graft position of query 1 (top, blue open circle) there are two annotated ancestral nodes from which query 1 inherits annotations, while for query 2 (bottom, blue open circle), there is only one annotated ancestral node and only the annotations from this one node are inherited by query 2

Here we present a new tool, TreeGrafter, which extends the tree-based annotation inference model to sequences that are not in the annotated reference tree. TreeGrafter grafts a query sequence onto the reference phylogenetic tree. Like any other sequence in the tree, the query sequence will inherit annotations (including function annotations, family label annotations etc.) from its annotated ancestral nodes in the tree (Fig. 1).

## 2 Materials and methods

A detailed description of the TreeGrafter algorithm, and sources for annotations, are provided in Supplementary Material. Briefly, each query sequence is matched to a protein family using HMM scoring (Mi *et al.,* 2017); the sequence is added to the family multiple sequence alignment; and RAxML (Stamatakis, 2014) is used to graft the sequence to the annotated family tree. Annotations are inherited from the annotated nodes in the tree that are ancestral to the graft point. Note that ancestral nodes can be annotated with losses of function as well as gains; in the case of losses the given function is *not* inherited by its descendants.

## 3 Validation and results

### 3.1 Accuracy of tree grafting

We performed leave-one-out testing to assess the ability of TreeGrafter to graft a sequence to the correct tree position, using eight complete proteomes across kingdoms and phyla (Supplementary Table S1). For each sequence, we first remove it from the corresponding PANTHER phylogenetic tree and multiple sequence alignments, and then graft the input sequence back to the reduced tree using TreeGrafter.

TreeGrafter outperformed subfamily HMM scoring (the standard used in PANTHER and InterProScan for nearly 20 years) for assigning sequences to the proper subfamily (Supplementary Table S1). This test was particularly stringent as we removed the validation sequences from the reference trees (and alignment), but not from the alignments used to train the subfamily HMMs. Using HMMER3 rather than MAFFT for the alignment step substantially increases speed (Supplementary Fig. S1) and also marginally increases performance on our subfamily classification benchmark.

### 3.2 Comparing GO annotations from TreeGrafter with InterPro2GO

Interpro2GO (Burge *et al.*, 2012) is the state-of-art and one of the most widely used tools for protein sequence annotation. InterPro signatures (primarily HMMs, including PANTHER) have been annotated with GO terms by expert curation. We compared the GO annotations from TreeGrafter and InterPro2GO for each protein sequence of the eight species (Supplementary Table S2). Overall, we find that for annotated proteins, TreeGrafter infers a larger number of GO annotations than InterPro2GO. When GO terms from the two methods are related in the GO hierarchy (and hence comparable), TreeGrafter annotations tend to be more specific. However, GO annotations from TreeGrafter do not completely overlap with InterPro2GO, and do not currently cover as many proteins, demonstrating the complementarity of the approaches. TreeGrafter will be incorporated into InterProScan in the near future, and the number of proteins annotated by TreeGrafter will continue to increase as the GO Phylogenetic Annotation project proceeds.

### 3.3 Limitations of TreeGrafter

Users should be aware of potential limitations of TreeGrafter that apply to phylogenetic methods in general. First, the results will depend on the accuracy of the input multiple sequence alignment, and the input reference tree. In our implementation, TreeGrafter uses the trees in PANTHER, which are reviewed and improved as part of the manual annotation process, but like any computational inference result, they can be incorrect. This can be particularly true for short proteins, or families with relatively high levels of sequence divergence. Second, the results will depend on how closely related a query sequence is to the sequences in the reference tree. Distant enough relationships can lead to the well-known ‘long branch attraction’ effect that will tend to graft distantly related sequences onto longer branches in the reference tree. Domain shuffling can also cause problems; in some cases of multi-domain families, the tree will be estimated based only on a single domain, which can lead to incomplete or even incorrect functional predictions.

## 4 Implementation

TreeGrafter is implemented in Perl as a standalone command line tool, available at https://github.com/pantherdb/TreeGrafter. To simplify installation, this repository also includes instructions for deploying the TreeGrafter Docker container.

## Supplementary Material

Supplementary Materials
