# Supplementary material for: TreeGrafter: phylogenetic tree-based annotation of proteins with Gene Ontology terms and other annotations
Source: Bioinformatics. 2018 Jul 19;35(3):518–20. doi: 10.1093/bioinformatics/bty625 (PMC6361231; doi:10.1093/bioinformatics/bty625)
Supplement: Supplementary Materials [file bty625_supplemental_materials.docx]

**Supplemental Methods**

**2.1 Trees, alignments and GO annotated gene trees from PAINT**

Sequence alignments and phylogenetic trees, and annotated subfamily nodes were obtained from the PANTHER database, version 12.0. GO annotations for nodes in the gene trees (Gaudet, et al., 2011) were obtained from the Gene Ontology github repository. Currently, approximately one third (4650/14710) of the PANTHER families have been curated with GO annotations.

**2.2 TreeGrafter algorithm: grafting and in-heritance of annotations**

Our grafting approach is similar to the TreeFam “orthology on the fly” tool (Schreiber, et al., 2014), but differs in how alignments are computed and how the graft point is determined. Pairwise alignments between a sequence and profile HMM (generated using hmmscan from HMMER3) are used to add the query sequence to a precomputed multiple alignment (produced by aligning the tree sequences to the profile HMM), rather than MAFFT (Katoh and Standley, 2013), which was found to be the rate-limiting step in most cases.

The grafting of a new sequence onto the reference tree proceeds in three steps: (i) the query is scored against the PANTHER HMM library (family HMMs only) to find a best matching reference phylogenetic tree (Mi et al. 2017) and to obtain an alignment to the family HMM; (ii) the alignment to the HMM is used to add the query to the pre-calculated multiple sequence alignment; (iii), RAxML version 8 (using parsimony mode for efficiency) is used to graft the query sequence onto the reference tree with the extended alignment that contains all sequences (reference + query) as input. If multiple, equally parsimonious graft points are found, their last common ancestor in the tree is used as the consensus graft point. The annotations of the graft point are obtained through a propagation process: annotations are inherited from parent node to descendant nodes in the path from the root of the reference tree to the graft point. In this process, ancestral gain of function annotations are inherited, and ancestral loss of function annotations prevent propagation.

**Additional References**

Katoh, K. and Standley, D.M. MAFFT multiple sequence alignment software version 7: improvements in performance and usability. *Molecular biology and evolution* 2013;30(4):772-780.

Schreiber, F.*, et al.* TreeFam v9: a new website, more species and orthology-on-the-fly. *Nucleic acids research* 2014;42(Database issue):D922-925.

**Supplemental Table 1. Comparison of TreeGrafter (using either MAFFT or HMMER3 for the multiple alignment step) with PANTHER subfamily HMM scoring tool**

| Species | Fraction of genes assigned to correct subfamily using **subfamily HMMs** | Fraction of genes assigned to correct subfamily using **TreeGrafter with MAFFT** | Fraction of genes assigned to correct subfamily using **TreeGrafter with HMMER3** |
| --- | --- | --- | --- |
| *Equus caballus* (Horse) | 0.912 | [0.941](file://localhost/tel/(940)%20825-6881) | 0.944 |
| *Homo sapiens* (Human) | 0.912 | [0.951](file://localhost/tel/(951)%20208-4363) | 0.955 |
| *Anolis carolinensis* (Green anole lizard) | [0.878](file://localhost/tel/(878)%20420-1622) | 0.890 | 0.893 |
| *Anopheles gambiae* (African malaria mosquito) | 0.823 | 0.898 | 0.905 |
| *Populus trichocarpa* (Western balsam poplar) | [0.830](file://localhost/tel/(829)%20677-7771) | [0.916](file://localhost/tel/(915)%20747-6434) | 0.913 |
| *Cryptococcus neoformans JEC21* (Fungus) | [0.855](file://localhost/tel/(854)%20935-0917) | [0.878](file://localhost/tel/(877)%20807-5417) | 0.886 |
| *Methanosarcina acetivorans* (Archaeon) | 0.841 | [0.854](file://localhost/tel/(854)%20259-1845) | 0.858 |
| *Salmonella typhimurium LT2* (Bacterium) | [0.912](file://localhost/tel/(912)%20458-9184) | 0.941 | [0.941](file://localhost/tel/(941)%20440-0956) |

**Supplemental Table 2. Comparisons between TreeGrafter and InterPro2GO annotations**

|  | **Protein sequences annotated in each species** | | | | **Annotations from TreeGrafter and InterPro2GO** | | | | **Annotations related in GO structure** | |
| --- | --- | --- | --- | --- | --- | --- | --- | --- | --- | --- |
| Species | Total number of protein sequences | Fraction of sequences with >=1 TreeGrafter annotation | Fraction of sequences with >=1 InterPro2GO annotation | Fraction of sequences with both InterPro2GO and TreeGrafter annotations | Total annotations from TreeGrafter | Total annotations from InterPro2GO | Identical annotations from InterPro2GO and TreeGrafter | Related but not identical annotations from InterPro2GO and TreeGrafter | Fraction of related annotations where TreeGrafter is more specific than InterPro2GO | Fraction of related annotations where InterPro2GO is more specific than TreeGrafter |
| *Equus caballus* (Horse) | 20308 | 0.448 | 0.733 | 0.356 | 33086 | 24006 | 2643 | 2989 | 0.846 | 0.154 |
| *Homo sapiens* (Human) | 21002 | 0.41 | 0.746 | 0.349 | 34480 | 23940 | 2502 | 3064 | 0.893 | 0.107 |
| *Anolis carolinensis* (Green anole lizard) | 18492 | 0.386 | 0.752 | 0.328 | 29716 | 19733 | 2030 | 2675 | 0.917 | 0.083 |
| *Anopheles gambiae* (African malaria mosquito) | 11625 | 0.36 | 0.668 | 0.301 | 15084 | 10408 | 1174 | 1552 | 0.929 | 0.071 |
| *Populus trichocarpa* (Western balsam poplar) | 41259 | 0.324 | 0.59 | 0.263 | 40513 | 30935 | 3317 | 4533 | 0.955 | 0.045 |
| *Cryptococcus neoformans JEC21* (Fungus) | 6602 | 0.405 | 0.622 | 0.326 | 9186 | 6658 | 745 | 962 | 0.964 | 0.036 |
| *Methanosarcina acetivorans* (Archaeon) | 4296 | 0.158 | 0.494 | 0.139 | 1754 | 1981 | 223 | 254 | 0.913 | 0.087 |
| *Salmonella typhimurium LT2* (Bacterium) | 4519 | 0.291 | 0.696 | 0.264 | 3499 | 4376 | 439 | 587 | 0.917 | 0.083 |


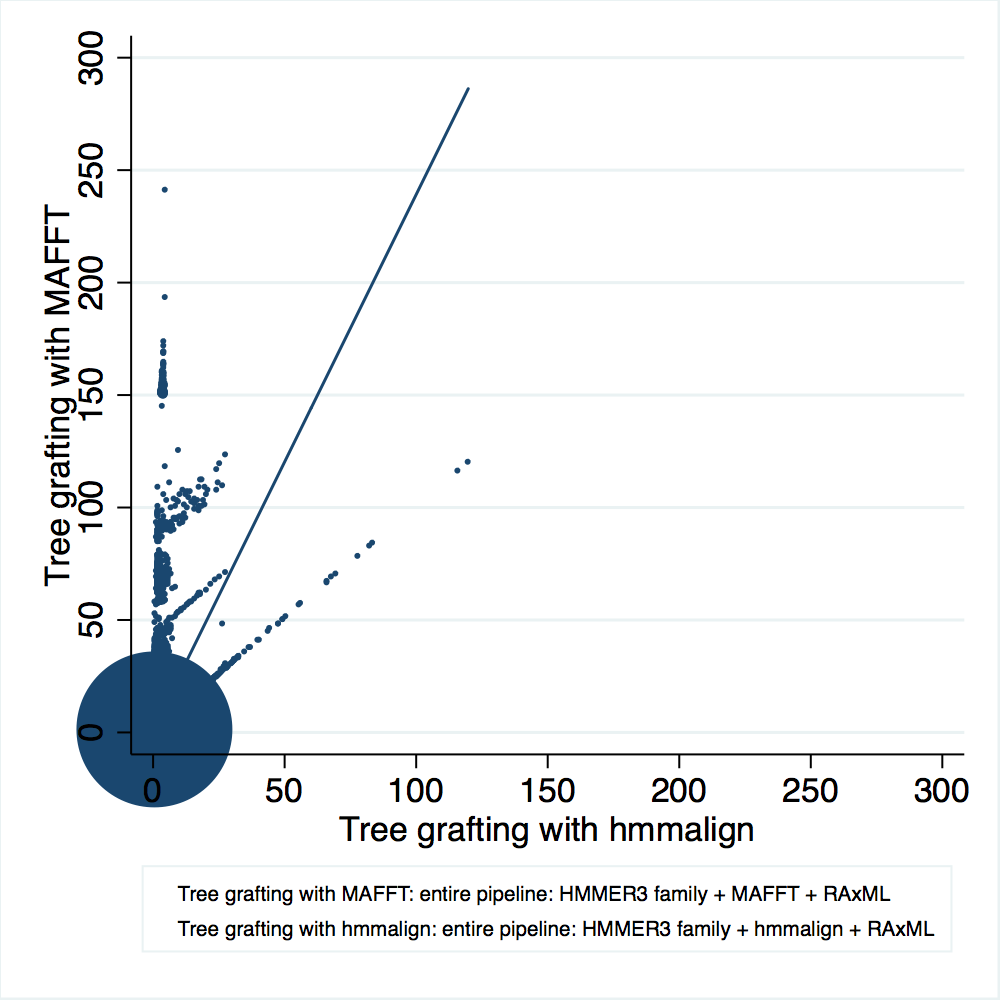

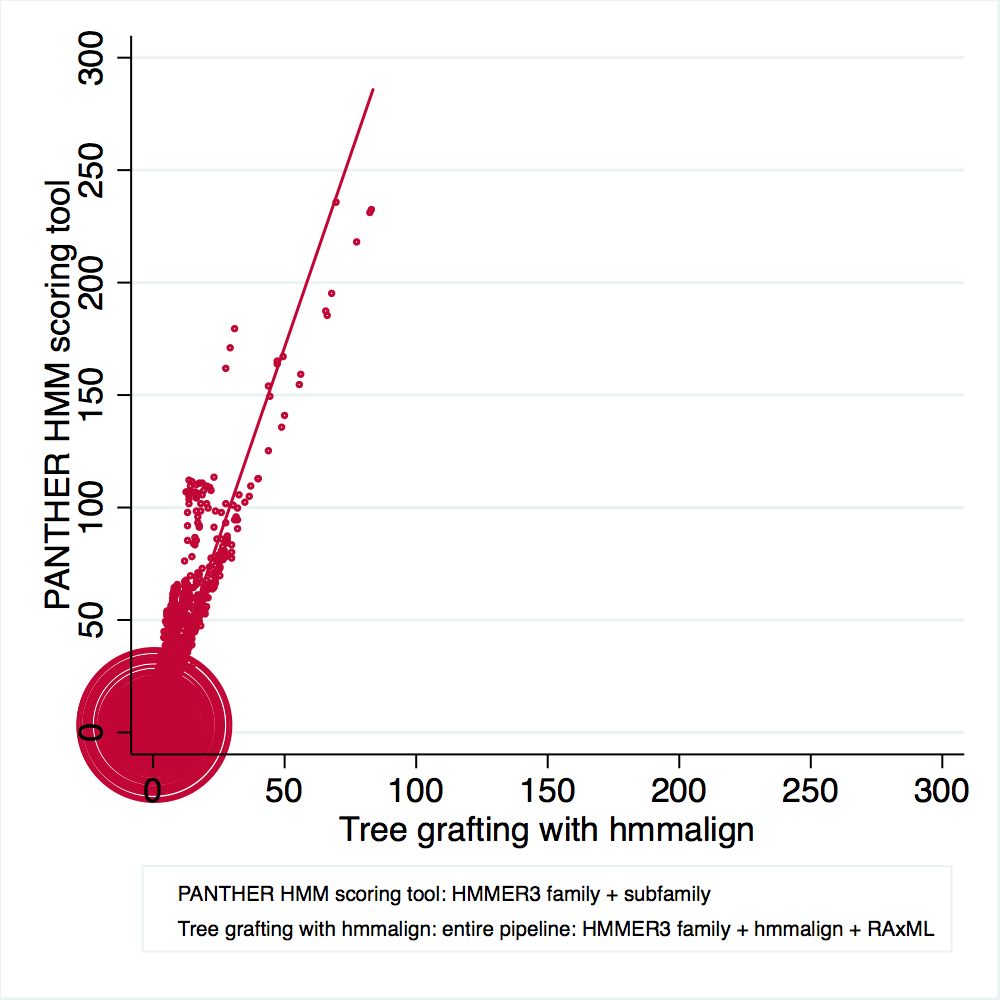


**Supplemental Figure 1. Run-time analysis.**

**Left panel: TreeGrafter runs faster using HMMER3 hmmalign, than with MAFFT, for the MSA step.** The radius of blue circles represents the number of overlapping points. Tree grafting with MAFFT (entire pipeline, HMMER3 family + MAFFT + RaxML) (y-axis) vs. Tree grafting with hmmalign (entire pipeline, HMMER3 family + hmmalign + RaxML) (x-axis).

**Right panel: TreeGrafter runs faster than the standard subfamily HMM scoring tool currently in PANTHER and InterProScan.** The radius of red circles represents the number of overlapping points. HMMER family+subfamily (y-axis) vs. Tree grafting with hmmalign (entire pipeline, HMMER3 family + hmmalign + RaxML) (x-axis).
